# Microwave-Assisted versus Conventional Isolation of Glucosinolate Degradation Products from *Lunaria annua* L. and Their Cytotoxic Activity

**DOI:** 10.3390/biom10020215

**Published:** 2020-02-01

**Authors:** Ivica Blažević, Azra Đulović, Vedrana Čikeš Čulić, Marijana Popović, Xavier Guillot, Franko Burčul, Patrick Rollin

**Affiliations:** 1 Department of Organic Chemistry, Faculty of Chemistry and Technology, University of Split, Ruđera Boškovića 35, 21000 Split, Croatia; azra@ktf-split.hr (A.Đ.); mpopovic@ktf-split.hr (M.P.); 2 School of Medicine, University of Split, Šoltanska 2, 21000 Split, Croatia; vcikesc@mefst.hr; 3 Laboulet Ets, 81500 Lavaur, France; xguillot@laposte.net; 4 Department of Analytical Chemistry, Faculty of Chemistry and Technology, University of Split, Ruđera Boškovića 35, 21000 Split, Croatia; franko@ktf-split.hr; 5 Institut de Chimie Organique et Analytique (ICOA), Université d’Orléans et CNRS, UMR 7311, BP 6759, F-45067 Orléans, France; patrick.rollin@univ-orleans.fr

**Keywords:** *Lunaria annua*, glucosinolates, isothiocyanates, microwave-assisted isolation, cytotoxic activity, human lung cancer cell line A549, breast cancer cell line MDA-MB-231

## Abstract

Glucosinolates (GSLs) from *Lunaria annua* L. seeds were analyzed qualitatively and quantitatively by their desulfo counterparts using UHPLC-DAD-MS/MS technique and by their volatile breakdown products, isothiocyanates (ITCs), using GC-MS technique. GSL breakdown products were obtained by conventional techniques (hydrodistillation in a Clevenger type apparatus (HD), CH_2_Cl_2_ extraction after myrosinase hydrolysis (EXT) for 24 h) as well as by modern techniques, microwave-assisted distillation (MAD) and microwave hydrodiffusion and gravity (MHG). Seven GSLs were identified as follows: isopropyl GSL (**1**), *sec*-butyl GSL (**2**), 5-(methylsulfinyl)pentyl GSL (**3**), 6-(methylsulfinyl)hexyl GSL (**4**), 5-(methylsulfanyl)pentyl GSL (**5**), 6-(methylsulfanyl)hexyl GSL (**6**), and benzyl GSL (**7**). Additionally, pent-4-enyl- and hex-5-enyl ITCs were detected in the volatile extracts. However, their corresponding GSLs were not detected using UHPLC-DAD-MS/MS. Thus, they are suggested to be formed during GC-MS analysis via thermolysis of 5-(methylsulfinyl)pentyl- and 6-(methylsulfinyl)hexyl ITCs, respectively. Volatile isolates were tested for their cytotoxic activity using MTT assay. EXT and MHG showed the best cytotoxic activity against human lung cancer cell line A549 during an incubation time of 72 h (IC_50_ 18.8, and 33.5 μg/mL, respectively), and against breast cancer cell line MDA-MB-231 after 48 h (IC_50_ 6.0 and 11.8 μg/mL, respectively). These activities can be attributed to the ITCs originating from **3** and **4**.

## 1. Introduction

*Lunaria annua* L. (annual honesty) belongs to the Brassicaceae family, which is, without exception, characterized by the presence of glucosinolates (GSLs) [1]. Native to Southeast Europe and Southwest Asia, *L. annua* is a biennial plant which flowers in mid-summer, developing its distinctive silver-dollar sized silicles that become translucent with maturity.

From the beginning, seeds of *L. annua* have served as a source of isopropyl isothiocyanate (ITC), originating from glucoputranjivin (**1**) degradation. Kjær (1959) first isolated **1** in the form of its crystalline tetraacetate [2]. In addition to **1**, using thin-layer chromatography, Danielak and Borkowski (1969) also reported glucocochlearin (**2**), and glucoberteroin (**5**) [3]. Using GC-MS analyses, Daxenbichler (1991) reported isopropyl ITC, *sec*-butyl ITC, 5-(methylsulfinyl)pentyl ITC, 6-(methylsulfinyl)hexyl ITC, and 5-(methylsulfanyl)pentyl ITC, originating from the corresponding **1**, **2,** glucoalyssin (**3**), glucohesperin (**4**), and **5**, respectively [4]. Seeds from wild-growing *L. annua* were used by Blažević et al. (2014) to investigate different influences (enzymatic, chemical, and thermal) affecting GSLs degradation. By studying their breakdown products, twelve GSLs were identified, among which **1**, **3, 4,** pent-4-enyl and hex-5-enyl GSLs dominated [5]. The latter GSL structure was not confirmed by the required spectrometry techniques (MS, NMR) [6]. Moreover, a most recent review in the field of GSLs revealed that only 88 of 137 GSLs found in plant kingdom were fully characterized by modern spectroscopy techniques up to mid-2018s [7]. The authors emphasized the critical aspects that should be considered in the practical use of GC-MS for GSL identification, due to the reactivity of ITCs and, in some cases, to thermal instability [7]. Bennett et al. reported that *L. annua* seeds contain 25 to 50 µmol/g dry weight (DW) of **1**, 0.1 to 10 µmol/g DW of **4** and **5**, and traces of glucolesquerellin (**6**) [8]. Quantization of GSLs is also an important piece of the puzzle that should be investigated in order to identify the sources of specific GSLs that could act as precursors of ITCs with designated biological activities [1,9,10]. The consumption of cruciferous vegetables is closely correlated to a lowered incidence of cancer, mostly due to ITCs. Although such beneficial activities of ITCs are primarily attributed to the -N=C=S functional group, it is also recognized that the type of the side chain strongly influences their properties, such as lipophilicity of the compound and electrophilicity of the ITC functional carbon atom [11].

As a part of our ongoing investigation of the conditions affecting GSLs degradation, microwaves were applied during distillation and extraction processes and compared to the conventional techniques, i.e., hydrodistillation in Clevenger type apparatus and CH_2_Cl_2_ extraction after myrosinase hydrolysis. GSLs of *L. annua* seeds were analyzed qualitatively and quantitatively using UHPLC-DAD-MS/MS analysis of desulfoglucosinolates (dGSLs) and GC-MS analysis of their breakdown volatiles. The obtained volatile isolates were assessed by MTT for their cytotoxic activities against human lung cancer cell line A549 and breast cancer cell line MDA-MB-231.

## 2. Material and Methods

### 2.1. Materials and Reagents

*Lunaria annua* L. seeds were obtained from cultivated plants (Laboulet Semences, Airaines, France). The voucher specimen (ZOKLA001) was deposited at the Department of Organic Chemistry, Faculty of Chemistry and Technology, Split, Croatia. Myrosinase and sinigrin were obtained from Sigma-Aldrich and glucotropaeolin was obtained from Phytoplan (Germany). All other chemicals and reagents were of analytical grade. Cancer cell lines (human lung cancer cell line A549 and breast cancer cell line MDA-MB-231) were cultured in a humidified atmosphere with 5% CO_2_ at 37 °C, in Dulbecco′s modified Eagle medium (DMEM, EuroClone, Milano, Italy) containing 4.5 g/L glucose, 10% fetal bovine serum (FBS), and 1% antibiotics (penicillin and streptomycin, EuroClone, Milano, Italy).

### 2.2. Isolation and Chemical Analysis

#### 2.2.1. Isolation of Desulfoglucosinolates

GSLs were extracted as previously reported [12]. Seeds were ground to a fine powder, from which 100 mg were extracted for 5 min at 80 °C in 2 × 1 mL MeOH/H_2_O (70:30 *v*/*v*) to inactivate the endogenous myrosinase. Each extract (1 mL) was loaded onto a mini-column filled with 0.5 mL of DEAE-Sephadex A-25 anion-exchange resin (GE Healthcare) conditioned with 25 mM acetate buffer (pH 5.6). After washing the column with 70% MeOH and 1 mL of ultrapure water, optimal conditions for desulfation were set by adding buffer solution. The mini-column was loaded with 20 μL (0.35 U/mL) of purified sulfatase, and left to stand overnight at room temperature. The dGSLs were then eluted with 1.5 mL of ultrapure H_2_O and the samples were stored at −20 °C until further analysis.

#### 2.2.2. HPLC-DAD Analysis of Desulfoglucosinolates

Analysis was performed on HPLC-DAD (Ultimate 3000, Thermo Fischer Scientific, USA) using Hypersil GOLD column (5 µm, 250 mm × 4.0 mm, Thermo Fischer Scientific, USA). Each extract was analyzed twice. A gradient consisting of solvent A (H_2_O) and solvent B (acetonitrile:H_2_O 30:70 *v*/*v*) was applied at a flow rate of 0.8 mL/min as follows: 0.14 min 96% A and 4% B, 28.14 min 14% A and 86% B, 32.14 min 14% A and 86% B, 34.14 min 5% A and 95% B, 47.14 min 5% A and 95% B, 48.14 min 96% A and 4% B, and 56.14 min 96% A and 4% B. The column temperature was held at 30 °C and the injection volume was 20 µL. For confirmation of the peaks from HPLC-DAD, individual peaks were subjected to UHPLC-MS/MS analysis.

The amount of GSLs was quantified using a calibration curve of pure desulfosinigrin solution (range from 0.14 to 1.4 mM) and RPFs for each individual dGSL [13]. RPF values for quantification of dGSLs were as following: RPF 1.0 for **1**, **4,** and **6 [14]**; RPF 1.07 and 0.95 for **3** and **7**, respectively [15]; and arbitrary RPF 1.0 for **2** and **5**.

#### 2.2.3. UHPLC-MS/MS Analysis

Analysis was performed on UHPLC-MS/MS (Ultimate 3000RS with TSQ Quantis MS/MS detector, Thermo Fischer Scientific, USA) using Hypersil GOLD column (3.0 µm, 3.0 × 100 mm, Thermo Fischer Scientific, USA). A gradient consisting of solvent A (50 μM NaCl in H_2_O) and solvent B (acetonitrile/H_2_O 30:70 *v*/*v*) was applied at a flow rate of 0.5 mL/min as follows: 0.14 min 96% A and 4% B, 7.84 min 14% A and 86% B, 8.96 min 14% A and 86% B;,9.52 min 5% A and 95% B, 13.16 min 5% A and 95% B, 13.44 min 96% A and 4% B, and 15.68 min 96% A and 4% B. The column temperature was held at 40 °C and the injection volume was 5 µL. The electrospray interface was H-ESI source operating with a capillary voltage of 3.5 kV at 350 °C. The system was operated in the positive ion electrospray mode.

#### 2.2.4. Isolation of Volatiles

##### Conventional Isolation

The volatiles were isolated by the following two approaches: Hydrodistillation in Clevenger type apparatus for 2.5 h using 50 g of ground seeds (HD), and CH_2_Cl_2_ extraction after endogenous and exogenous hydrolysis by myrosinase (1 to 2 units) for 24 h at 27 °C using 10 g of ground seeds (EXT), as described previously [12,16].

##### Microwave-Assisted Isolation

Seeds were hydrated for 2 h prior to the isolation process. A Milestone ‘ETHOS X’ microwave laboratory oven (1900 W maximum) was used for microwave-assisted isolation. This is a multimode microwave reactor of 2.45 GHz. Temperature is monitored by an external infrared sensor.

**Figure 1:**
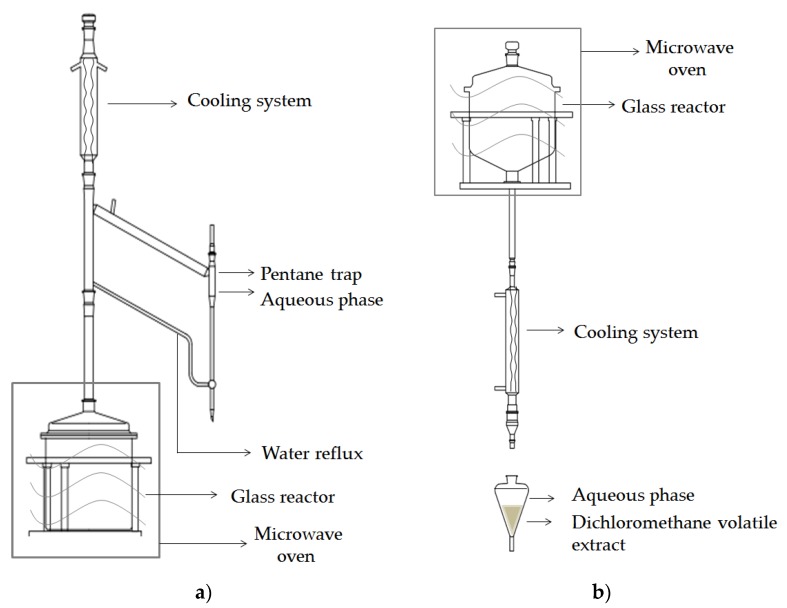
Scheme of the apparatus for microwave-assisted isolation. (**a**) Microwave-assisted distillation (MAD); (**b**) microwave hydrodiffusion and gravity (MHG).

Microwave-assisted distillation (MAD): A typical experiment is conducted at atmospheric pressure with 100 g of matrix, during 35 min at 500 W (98 °C). The distillation process started after 10 min. The distillate was collected in a side-tube using pentane trap, dried over anhydrous sodium sulphate, and stored at −20 °C, until analysis (Figure 1a).

Microwave hydrodiffusion and gravity (MHG): A typical experiment is conducted at atmospheric pressure with 100 g of matrix, during 15 min at 500 W (98 °C). The water extract was collected and extracted by CH_2_Cl_2_, dried over anhydrous sodium sulphate and concentrated using an automated sample concentrator (VLM GmbH, Germany) to a volume of 1 mL. The sample was stored at −20 °C, until analysis (Figure 1b).

#### 2.2.5. GC-MS Analysis

All isolates were analyzed by GC-MS (model 3900-2100T; Varian Inc., Lake Forest, CA, USA) using a VF-5MS column (30 m × 0.25 mm i.d., coating thickness 0.25 μm) programmed at 60 °C isothermal for 3 min, increased to 246 °C at a rate of 3 °C/min and held isothermal for 25 min, as previously described [12]. The analyses were carried out in duplicate.

Individual peaks of volatiles were identified by comparing their retention indices and mass spectra with those of authentic samples, as well as by computer matching against the Wiley 7 spectral database and comparison of the mass spectra with literature data [17,18,19]. The analyses were run in duplicate and the percentages in Table 2 were calculated as the mean value of component percentages on a VF-5MS column.

### 2.3. Cell Viability Assay (MTT)

MTT spectrophotometric assay was performed on a microplate photometer, model HiPo MPP-96 (BioSan, Riga, Latvia), as previously described [10,12]. The cells (A549 and MDA-MB-231) were treated with *L. annua* volatile isolates (HD, EXT and MHG) at concentrations of 1, 5, 10, 50, and 100 µg/mL in a complete medium (in triplicate) for 4, 24, 48, and 72 h. In order to determine IC_50_ more accurately, both cell lines were treated with volatile isolates at additional concentrations. The A549 cell line was treated with 20, 30, and 40 µg/mL concentrations of EXT and MHG. The MDA-MB-231 cell line was treated with 2.5, 7.5, and 30 μg/mL concentrations of EXT and with 20, 30, and 40 µg/mL concentrations of MHG. After treatment with the isolated compounds, the cells were incubated with 0.5 g MTT/L at 37 °C for 2 h, followed by removal of the medium. Dimethyl sulfoxide (DMSO) was added and incubated for another 10 min at 37 °C while shaking. The degree of formazan formation, an indicator of living and metabolically active cells, was measured at 570 nm. The data were calculated in relation to the untreated control (100%) from three independent measurements. The calculation of IC_50_ values was performed using GraphPad Prism software version 7.0. The criteria used to categorize the activity against the tested cell lines was based on IC_50_ values as follows: 20 μg/mL = highly active, 21 to 200 μg/mL = moderately active, 201 to 500 μg/mL = weakly active, and >501 μg/mL = inactive [20].

## 3. Results and Discussion

### 3.1. Glucosinolates and Volatile Constituents

**Figure 2:**
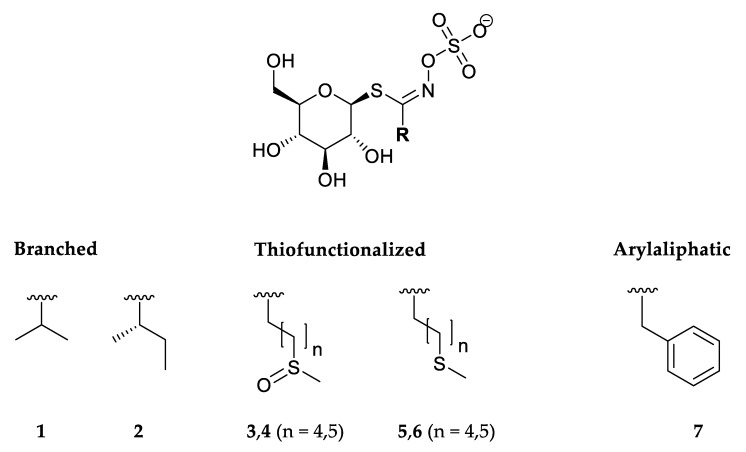
Structures of the glucosinolates identified in *Lunaria annua* seeds. **1**—Glucoputranjivin, **2**—glucocochlearin, **3**—glucoalyssin, **4**—glucohesperin, **5**—glucoberteroin, **6**—glucolesquerellin, **7**—glucotropaeolin.

GSLs of *Lunaria annua* seed were qualitatively analyzed by UHPLC-MS/MS and quantified by HPLC-DAD of their desulfo counterparts (Table 1, Figure 2, Figures S1 and S2). GSLs were subjected to enzymatic and thermal degradation. Enzymatic degradation was performed by endogenous and exogenous myrosinase, whereas thermal degradation was assessed using conventional (hydrodistillation) and up-to-date (MAD and MHG) techniques. Isothiocyanates (ITCs), the main volatiles that originate from degradation of GSLs, were identified by GC-MS. (Table 2).

Seven GSLs were detected by UHPLC-DAD-MS/MS analyses. The structures of the corresponding GSLs are shown in Figure 2.

Next to dGSL sodium adduct, MS^2^ showed characteristic fragments *m*/*z* = 185 or 219 corresponding to [C_6_H_10_O_5_ + Na]^+^ and [thioGlc + Na]^+^, respectively (Figure S2). Two branched GSLs **1,** and **2** originated from Val and Ile biosynthetic pathway, respectively. The most abundant peak observed at *t*_R_ = 3.80 min showed characteristic dGSL sodium adduct *m*/*z* = 304 which was identified as desulfoglucoputranjivin (**d1**), with 14.60 μmol/g of DW. Isopropyl ITC originating from **1** was found in all volatile samples obtained by different isolation methods, ranging from 35% to 92%. dGSL sodium adduct of **2** *m*/*z* = 318 was detected at *t*_R_ = 5.34 min. GSL **2** and its regioisomer isobutyl GSL, both known to exist in plants, cannot be distinguished by MS^2^ spectra [21]. However, MS spectra of the corresponding ITCs, having the same odd molecular ion *m*/*z* = 115 and characteristic *m*/*z* = 72 [CH_2_NCS]^+^, can be easily discriminated as *sec*-butyl ITC delivers a strong *m*/*z* 86 fragment in difference to isobutyl ITC [17] that enabled us to ascertain the structure of **2**.

**Figure 3:**
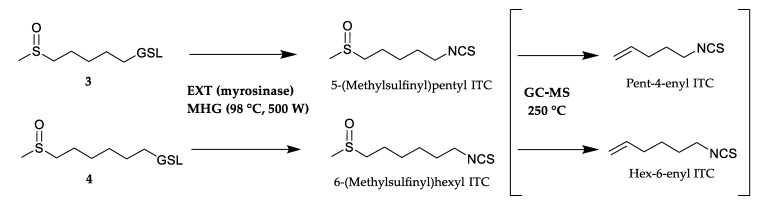
Degradation of 5-(methylsulfinyl)pentyl GSL (**3**) and 6-(methylsulfinyl)hexyl GSL (**4**) to methylsulfinylalkyl isothiocyanates (ITCs) products present in CH_2_Cl_2_ extraction after myrosinase hydrolysis (EXT) and microwave hydrodiffusion and gravity (MHG) and their thermolysis products during GC-MS analysis (not present in EXT and MHG).

Four thiofunction-containing GSLs (**3**–**6**) originate from the Met biosynthetic pathway. The content of two ω-(methylsulfinyl)alkyl GSLs **3** and **4** were 1.74 and 3.54 μmol/g DW, while **5** and **6** were detected as traces. 5-(Methylsulfinyl)pentyl ITC (3.30%) and 6-(methylsulfinyl)hexyl ITC (2.31%) originating from **3** and **4**, respectively, were identified only in the volatile isolate obtained by CH_2_Cl_2_ extraction after myrosinase hydrolysis (EXT) in their native form. Two alkenyl ITCs, namely pent-4-enyl ITC (1.56 and 24.34%) and hex-5-enyl ITC (2.34 and 32.02%), were also detected in EXT and MHG seemingly indicating the putative presence of two more corresponding GSLs. However, no such alkenyl dGSLs were identified by UHPLC-MS/MS analysis. Thus, it may be suggested that pent-4-enyl ITC and hex-5-enyl ITC are formed through thermolysis of 5-(methylsulfinyl)pentyl ITC and 6-(methylsulfinyl)hexyl ITC, respectively, during GC-MS analysis (Figure 3).

These findings are in agreement with the study of Chiang et al. who reported that a standard solution of 4-(methylsulfinyl)butyl ITC (sulforaphane) analyzed by GC-MS showed ca. 80% thermal degradation to but-3-enyl ITC [22]. This was also previously reported in the case of *Arabis turrita* L. for C8-C10 alkyl GSLs bearing a terminal sulfoxide moiety on the side chain [23]. In summary, both EXT and MHG contained 5-(methylsulfinyl)pentyl ITC (27.64% and 1.56%, respectively) and 6-(methylsulfinyl)hexyl ITC (34.33% and 2.34%, respectively) while HD and MAD did not contain any of these ITCs.

Generally, the yields of the volatiles obtained by conventional techniques were much higher than those obtained by microwave-assisted isolation (Table 2). HD (100 °C, 2.30 h) which uses large quantities of water and energy in comparison to MAD and MHG, enabled degradation of GSLs and isolation of mostly low molecular and less polar ITCs (from **1**, **2**, and **5**–**7**). Volatiles obtained by MAD (500 W, 98 °C, 30 min) showed a similar profile of GSL degradation products as HD, albeit with 152-fold less yield (3.80 and 578.90 µg/g, respectively). GC-MS analysis revealed that the extraction of enzymatically formed GSLs breakdown volatiles enabled isolation of sulfoxide-bearing aliphatic ITCs, namely 5-(methylsulfinyl)pentyl ITC and 6-(methylsulfinyl)hexyl ITC. Those ITCs were not isolated by HD or MAD, probably due to their higher polarity.

### 3.2. Cytotoxic Activity

**Figure 4:**
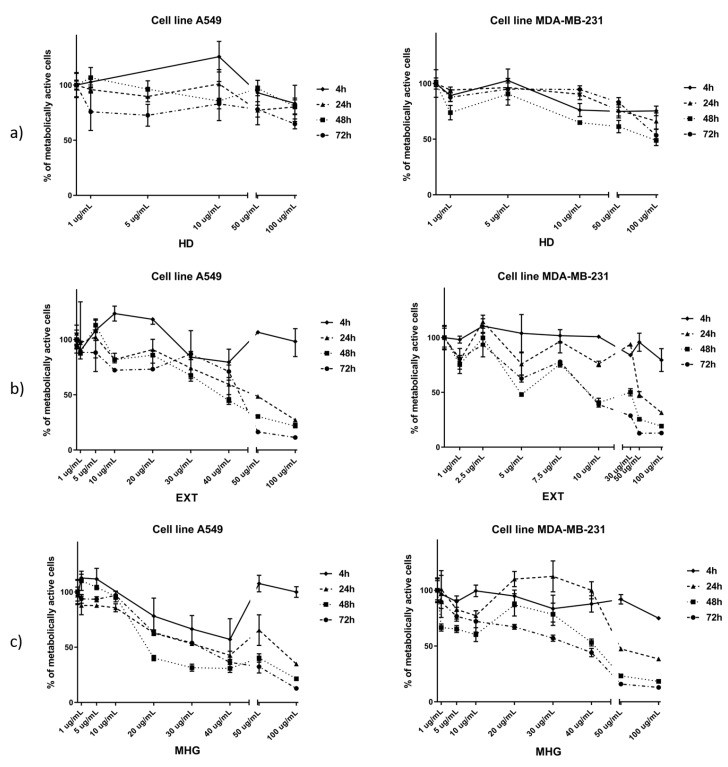
Percentage of metabolically active human lung cancer cell line A549 and breast cancer cell line MDA-MB-231 after 4, 24, 48, and 72 h of incubation with different concentrations of: (**a**) hydrodistillate in Clevenger type apparatus (HD), (**b**) CH_2_Cl_2_ extraction after 24 h of autolysis and added myrosinase (EXT), and (**c**) microwave hydrodiffusion and gravity (MHG). Calculated IC_50_ values (μg/mL) are given in Table S1.

Hydrodistillate (HD), CH_2_Cl_2_ extract after myrosinase hydrolysis (EXT), and microwave hydrodiffusion and gravity (MHG) were tested against human lung cancer cell line A549 and breast cancer cell line MDA-MB-231 for their cytotoxic activity (Figure 4) (Table S1). The criteria used to categorize the activity against the tested cell lines was based on IC_50_ values as described under Materials and Methods.

According to the IC_50_, the best cytotoxic activities against A549 cell line were observed for EXT and MHG. EXT showed the best activity after 72 h with IC_50_ of 39.15 μg/mL, while MHG showed the best activity after 48 h with IC_50_ of 26.79 μg/mL and both can be considered as moderately active. HD showed the lowest activity (IC_50_ not reached at 100 μg/mL).

When tested against the MDA-MB-231 cell line, the best cytotoxic activities were shown after 72 h. EXT showed to be highly active with IC_50_ 11.27, while MHG showed 2.3-fold lower activity, having IC_50_ 25.83 μg/mL.

In all volatile isolates, isopropyl ITC was found in highest percentage. HD isolate, having 92% of isopropyl ITC, showed the lowest activity on both cell lines tested. In contrast to EXT and MHG, HD did not contain either 5-(methylsulfinyl)pentyl- or 6-(methylsulfinyl)hexyl ITCs. Therefore, the high cytotoxic activities of EXT and MHG can be attributed to the presence of those two ITCs.

In recent years, 6-(methylsulfinyl)hexyl ITC has been widely studied as a major bioactive compound found in Japanese spice wasabi (*Wasabia japonica* (Miq.) Matsum.). In vivo and in vitro studies have demonstrated that 6-(methylsulfinyl)hexyl ITC has several biological properties regulated through diverse multiple pathways. Nomura et al. studied the effect of 6-(methylsulfinyl)hexyl ITC against several human cancer cell lines (HCC panel) and found the most effective suppression in low concentration towards breast cancer MDA-MB-231 and melanoma LOX-IMVI cell lines. They also compared the activity of similar compounds found in food, i.e., 4-(methylsulfinyl)butyl ITC (sulforaphane, from broccoli) and artificially synthesized ones, namely 2-(methylsulfinyl)ethyl and 8-(methylsulfinyl)octyl ITCs. All tested compounds suppressed cancer cell lines, however 6-(methylsulfinyl)hexyl ITC was the most effective at lower concentrations [24]. Fuke et al. also demonstrated the inhibitory effect of 6-(methylsulfinyl)hexyl ITC in human breast cancer cell line through promotion of apoptosis by inhibiting NF-κB and its control of the PI3K/AKT pathway [25].

Morroni et al. studied the neuroprotective effects of 6-(methylsulfinyl)hexyl ITC in a Parkinson′s disease mouse model and found that the decrease of apoptotic cell death and the activation of glutathione-dependent antioxidant systems could be underlying a mechanism of beneficial ITC effect [26]. Further investigation of the neuroprotective effect on IMR-32 human neuroblastoma cells was conducted by Trio et al., who concluded that 6-(methylsulfinyl)hexyl ITC can exert a neuroprotective effect by activating the Nrf2-mediated oxidative stress response pathway [27]. Likewise, 6-(methylsulfinyl)hexyl ITC showed inhibitory effect on colon carcinogenesis through a p53-independent mitochondrial dysfunction pathway [28]. Inhibition of viability, accompanied by the features of mitotic arrest and apoptosis of human pancreatic cancer cell lines PANC-1 and BxPC-3 were also observed for 6-(methylsulfinyl)hexyl ITC [29]. The latest study showed that 6-(methylsulfinyl)hexyl ITC inhibited the viability of human chronic myelogenous leukemia K562 cells, as well as suggesting its anti-leukemia activity [30].

These findings are in accordance with observed high cytotoxic activities of *L. annua* EXT and MHG against human lung cancer cell line A549 and breast cancer cell line MDA-MB-231 which can be associated with the presence of 5-(methylsulfinyl)pentyl ITC and 6-(methylsulfinyl)hexyl ITC.

## 4. Conclusions

The volatiles obtained from *L. annua* seeds by CH_2_Cl_2_ extraction after myrosinase hydrolysis showed high cytotoxic effect on the tested tumor cell lines. Such activity can be associated with the ITCs, which are present in large proportion. It can be suggested that the ω-(methylsulfinyl)alkyl ITCs are responsible for the observed high activities against the tested cancer cell lines, making them potential candidates for anticancer treatment studies. The compounds obtained by microwave hydrodiffusion and gravity were consistent with the ones obtained by the extraction after the enzyme hydrolysis. However, the yields obtained using the microwave techniques were much lower. Therefore, it is necessary to optimize time and power in order to produce the compounds of interest with improved yields.

## Figures and Tables

**Table 1:** Individual glucosinolate content (μmol/g DW) in *L. annua* seed.

	Glucosinolate (Trivial Name)	*t*_R_ (min)	Content (μmol/g DW)	[M + Na]^+^
***Branched***				
**1**	**Isopropyl GSL** (Glucoputranjivin)	3.80	14.60 ± 0.59	304
**2**	***sec-*Butyl GSL** (Glucocochlearin)	5.34	0.41 ± 0.11	318
***Sulfur-containing***				
**3**	**5-(Methylsulfinyl)pentyl GSL** (Glucoalyssin)	3.90	1.74 ± 0.13	394
**4**	**6-(Methylsulfinyl)hexyl GSL** (Glucohesperin)	5.05	3.54 ± 0.21	408
**5**	**5-(Methylsulfanyl)pentyl GSL** (Glucoberteroin)	7.61	Tr	378
**6**	**6-(Methylsulfanyl)hexyl GSL** (Glucolesquerellin)	9.11	Tr	392
***Arylaliphatic***				
**7**	**Benzyl GSL** (Glucotropaeolin)	6.06	1.54 ± 0.31	352
**Total** (μmol/g DW)			21.83 ± 1.35	

[M + Na]^+^, sodium adducts of desulfoglucosinolate; DW, dry weight; tr < 0.1 μmol/g DW, value is the mean ± standard error (*n* = 2).

**Table 2:** Degradation volatile products, their parent glucosinolates, and others identified by GC-MS of *L. annua* volatile isolates.

Parent Glucosinolate/ Identified Compound	RI	Conventional Techniques		Microwave-Assisted Isolation	
		HD	EXT	MAD	MHG
**Glucoputranjivin**					
Isopropyl ITC ^a,b,c^	858	92.13	35.56	87.03	88.02
**Glucocochlearin**					
*sec*-Butyl ITC ^a,b,c^	952	4.59	0.39	4.11	3.08
**Glucoalyssin**					
5-(Methylsulfinyl)pentyl ITC (alyssin) ^a,c^	2005	-	3.30	-	-
Pent-4-enyl ITC ^a,c, ⸸^	1093	-	24.34	-	1.56
**Glucohesperin**					
6-(Methylsulfinyl)hexyl ITC (hesperin) ^a,c^	2104	-	2.31	-	-
Hex-5-enyl ITC ^a,c, ‡^	1226	-	32.02	-	2.34
**Glucoberteroin**					
5-(Methylsulfanyl)pentyl ITC (berteroin) ^a,c^	1566	0.21	-	-	-
**Glucolesquerellin**					
6-(Methylsulfanyl)hexyl ITC (lesquerellin) ^a,c^	1714	0.29	-	-	-
**Glucotropaeolin**					
Benzyl ITC ^a,b,c^	1395	0.14	-	-	-
**Others**					
*S*-Methyl- methanethiosulfinate ^a,b,c^	1005	-	0.63	-	-
Diisopropyl disulfide ^a,b,c^	1010	-	0.10	0.33	0.14
Phenylacetaldehyde ^a,b,c^	1072	-	-	-	0.31
Hexadecanoic acid ^a,b,c^	1980	1.04	-	0.38	0.82
Total (%)		98.40	98.66	92.05	96.26
Yield (µg/g)		578.90	2331.20	3.80	47.10

HD, hydrodistillate in Clevenger type apparatus; EXT, CH_2_Cl_2_ extraction after 24 h of autolysis and added myrosinase; MAD, microwave-assisted distillation; MHG, microwave hydrodiffusion and gravity. RI, retention indices determined on a VF-5MS capillary column; -, not detected; tr, traces; ITC, isothiocyanate. ^a^ Compound identified by mass spectra and RI comparison with homemade library. ^b^ Compound identified by mass spectra comparison with Wiley library. ^c^ Compound identified by mass spectra comparison with literature values [17,18,19]. ^⸸^ Compound identified represents GC-MS thermal decomposition artefact of 5-(methylsulfinyl)pentyl ITC. ^‡^ Compound identified represents GC-MS thermal decomposition artefact of 6-(methylsulfinyl)hexyl ITC.

## Supplementary Materials

The following are available online at https://www.mdpi.com/2218-273X/10/2/215/s1, Figure S1: HPLC chromatogram of the desulfoglucosinolates of *L. annua* seeds. Numbers **1**–**7** refer to those given in Table 1, Figure S2: Fragments of sodium adducts of desulfated glucosinolates from *L. annua* observed in MS^2^ (collision energy 15V) after HPLC separation. **d1**–**d7** corresponds to desulfoglucosinolates in Table 1.
