# Supplementary material for: Microwave-Assisted versus Conventional Isolation of Glucosinolate Degradation Products from Lunaria annua L. and Their Cytotoxic Activity
Source: Biomolecules. 2020 Feb 1;10(2):215. doi: 10.3390/biom10020215 (PMC7072642; doi:10.3390/biom10020215)
Supplement: Supplementary file 1 [file biomolecules-10-00215-s001.pdf]

biomolecules

MDPI

Supplementary Materials (S1)

Article

Microwave-assisted- vs conventional isolation of glucosinolate degradation products from *Lunaria annua* L. and their cytotoxic activity

Ivica Blažević [1,*], Azra Đulović [1], Vedrana Čikeš Čulić [2], Marijana Popović [1], Xavier Guillot [3], Franko Burčul [4] and Patrick Rollin [5]

[1] Department of Organic Chemistry, Faculty of Chemistry and Technology, University of Split, Ruđera Boškovića 35, 21000 Split, Croatia; azra@ktf-split.hr (A.Đ.); mpopovic@ktf-split.hr (M.P.);

[2] School of Medicine, University of Split, Šoltanska 2, 21000 Split, Croatia; vcikesc@mefst.hr;

[3] Laboulet Ets, 81500 Lavaur, France; xguillot@laposte.net

[4] Department of Analytical Chemistry, Faculty of Chemistry and Technology, University of Split, Ruđera Boškovića 35, 21000 Split, Croatia; franko@ktf-split.hr

[5] Institut de Chimie Organique et Analytique (ICOA), Université d'Orléans et CNRS, UMR 7311, BP 6759, F-45067 Orléans, France; patrick.rollin@univ-orleans.fr

* Correspondence: blazevic@ktf-split.hr; Tel.: +385 21 329 434

Received: date; Accepted: date; Published: date

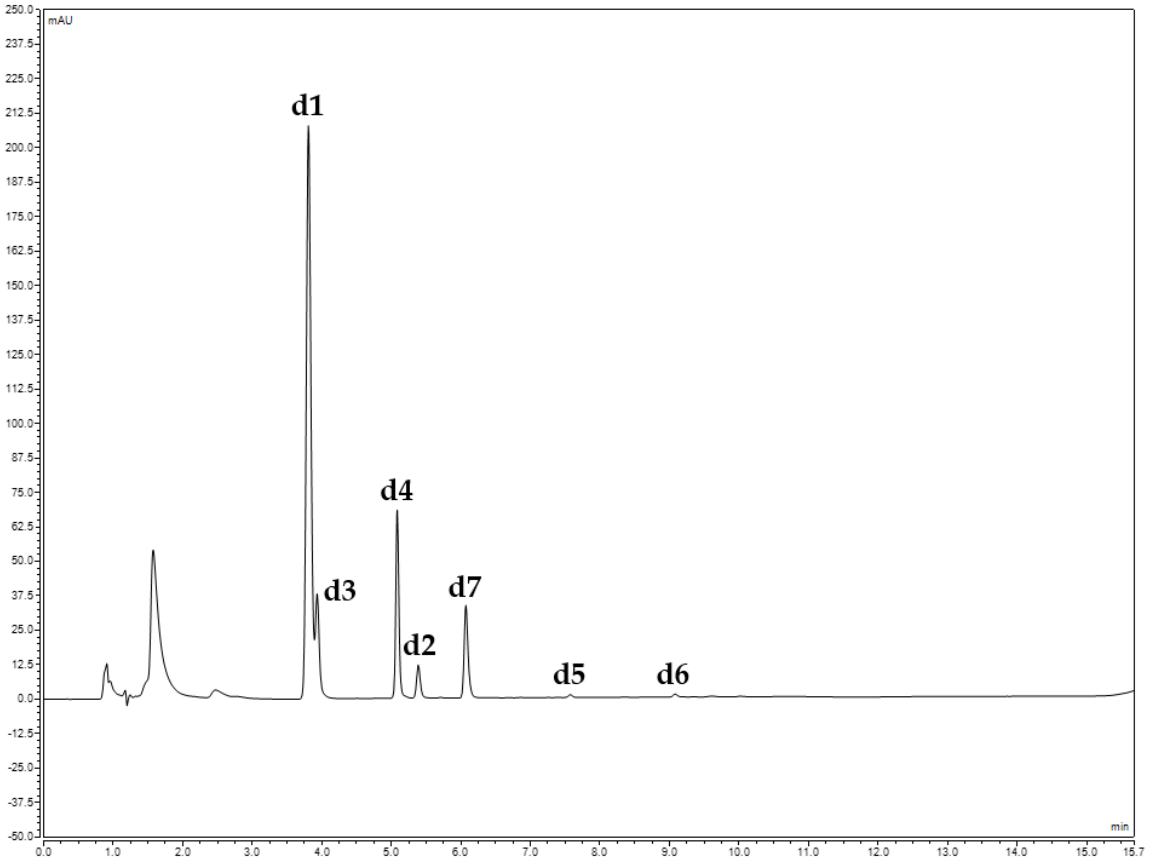

Figure S1. HPLC chromatogram of the desulfoglucosinolates of *L. annua* seeds. **d1** - desulfoglucoputranjivin, **d2** - desulfoglucocochlearin, **d3** - desulfoglucoalyssin, **d4** - desulfoglucohesperin, **d5** - desulfoglucoberteroin, **d6** - desulfoglucolesquerellin, **d7** - desulfoglucotropaeolin.

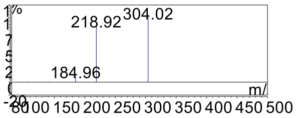

d1

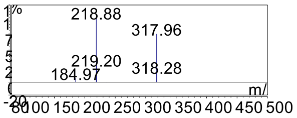

d2

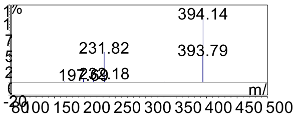

d3

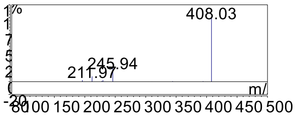

d4

Figure S2. *Continues*

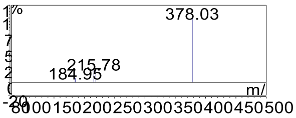

d5

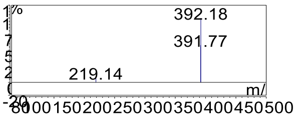

d6

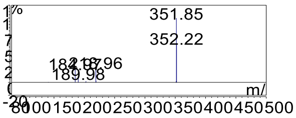

d7

Figure S2. Fragments of sodium adducts of desulfated glucosinolates from *L. annua* observed in MS^2 (collision energy 15V) after HPLC separation. **d1**–**d7** corresponds to desulfoglucosinolates in Figure S1.

Table S1. Calculated IC_{50} values (µg/mL) for volatiles obtained by HD, EXT and MHG from the seeds of *Lunaria annua* L. against human lung cancer cell A549 and breast cancer cell MDA-MB-231 lines after the 4, 12, 48 and 72h.

**Cell line**	**HD**				**EXT**				**MHG**			
	**4h**	**12h**	**48h**	**72h**	**4h**	**12h**	**48h**	**72h**	**4h**	**12h**	**48h**	**72h**
**A549**	>100.0	> 00.0	>100.0	>100.0	>100.0	44.53	33.20	18.79	>100.0	67.54	44.59	33.46
**MDA-MB-231**	>100.0	>100.0	67.44	>100.0	>100.0	37.10	6.00	7.10	>100.0	46.35	11.8	16.76
